# Incidence and Clinical Implications of Autoimmune Thyroiditis in the Development of Acne in Young Patients

**DOI:** 10.3390/diagnostics11050794

**Published:** 2021-04-28

**Authors:** Laura Endres, Delia Mirela Tit, Simona Bungau, Nicoleta Anamaria Pascalau, Laura Maghiar Țodan, Erika Bimbo-Szuhai, Gabriela Mariana Iancu, Nicoleta Negrut

**Affiliations:** 1 Department of Psycho-Neuroscience and Recovery, Faculty of Medicine and Pharmacy, University of Oradea, 410073 Oradea, Romania; laura_endres@yahoo.com (L.E.); n.pascalau@yahoo.com (N.A.P.); lnm_n10@yahoo.com (N.N.); 2 Department of Pharmacy, Faculty of Medicine and Pharmacy, University of Oradea, 410028 Oradea, Romania; mirela_tit@yahoo.com; 3 Doctoral School of Biomedical Sciences, University of Oradea, 410073 Oradea, Romania; 4 Department of Mofological Disciplines, Faculty of Medicine and Pharmacy, University of Oradea, 410073 Oradea, Romania; bszera@gmail.com; 5 Dermatology Department, Faculty of Medicine, “Lucian Blaga” University of Sibiu, 550169 Sibiu, Romania; mgabiancu@yahoo.com; 6 Clinic of Dermatology, County Emergency Hospital of Sibiu, 550245 Sibiu, Romania

**Keywords:** acne vulgaris, skin disorders, dermatology, autoimmune thyroiditis, hypothyroidism, hyperthyroidism

## Abstract

Autoimmune thyroiditis (AIT) is on the rise among the population, and is frequently associated with patients with acne vulgaris, especially females aged between 18–55 years old. The connection between the two is not fully elucidated. In this study, 236 patients diagnosed with acne in the dermatological office of the private Pelican Hospital and in few private dermatological offices from Oradea, Romania, during January 2018–December 2020, aged between 12 and 55 years old, were endocrinologically investigated to determine AIT and its influence on the severity of the acne. The values for the thyroid antibodies and thyroid-stimulating hormone (TSH) were determined for all of the subjects. The frequency of AIT in the study group was 72% and was associated with severe acne (*p* < 0.001). Patients with AIT with normal or hypofunction had more frequent severe acne than those with hyperfunction (*p* < 0.001, *p* = 0.002). The TSH and anti thyroidperoxidase (TPO) values did not influence the severity of the acne (*p* = 0.494; *p* = 0.111), while the anti-TG values were associated with severe acne (*p* = 0.007). The risk analysis indicated that raised values of anti-TPO (2.91 times greater) correlated with high anti-thyroglobulin (TG) values (4.36 times greater) doubled the risk of developing severe acne in patients. In acne evolution, the existence of AIT involves significant modifications.

## 1. Introduction

Young individuals from all around the world are affected by acne vulgaris (AV), a multifaceted skin disorder with a significant impact on quality of life, affecting more than 85% of young individuals [1]. Epidemiological studies indicate that it can occur at any age, despite the fact that it generally appears throughout puberty and is aggravated during adolescence [2]. The characteristics of acne vulgaris (AV) are comedones, secondary dyspigmentation, inflammatory lesions, and scarring, and this skin condition is relatively frequent [3].

There are multiple factors that determine this disease, among these, the four most pathogenous are high androgens levels determining pilosebaceous duct abnormal hyperkeratinization, resulting in comedones formation; high androgens levels that determine a rise in sebum production out of an increased sebaceous gland; *Propionilbacterium* (P.) acnes immunological activity, which determines an inflammatory reaction; and expansion and colonization of the duct with microorganisms usually with *P. acnes*, despite the fact that an interrelation connecting AV with *P. acnes* has not been demonstrated [3,4].

Besides the traditional view, which considers that acne is determined by the hyperplasia of the sebaceous glands, anomalous follicular differences with magnified keratinization, bacterial hypercolonization of the follicular ducts, and accentuated irritation mainly by triggering the adaptative immunity can contribute as well [5]. Regards the way in which chronic acne vulgaris develops, an important autoinflammatory function is presumed to be involved. There are autoinflammatory conditions reported to be linked to acne that could develop in the same context, entailing imbalanced immunity with atypical interleukin-1 signaling, determining considerable pathological inflammation from a clinical perspective [6]. In various chronic inflammatory skin disorders that comprise chronic idiopathic urticaria and acne vulgaris, thyroid autoimmunity has been discovered [7].

Autoimmune thyroiditis (AIT) may progress to normal thyroid function (in which case, asymptomatic patients have elevated specific antibody values), thyroid hypofunction (Hashimoto’s thyroiditis), or thyroid hyperfunction (Graves’ disease). Hashimoto thyroiditis has a prevalence of 0.3–1.5 cases per 1000 inhabitants, being the leading cause of hypothyroidism in American patients over 6 years of age, as well as in areas with a normal iodine intake [8,9]. Of those affected by the disease, 95% are women, and it has a descending transmission to first-degree relatives of patients with AIT. The age group between 30–50 years is more prone to this pathology [9]. Internationally, Graves’ disease accounts for 60–90% of thyrotoxicosis cases, with incidence ranging from 80–200 cases per 100,000 inhabitants. The condition is more common in young women aged between 30–60 years old [10].

The prevalence of AIT specific antibodies is 15–25% in the general population, is more common in women, and varies with age. The exact cause of the onset of the immune process against its own thyroid structures is unknown. To date, genetic factors (HLA DR-3 and T cell regulatory genes, such as CTLA 4), infectious factors, food (vitamin D, vitamin B12, selenium, and iodine), environmental factors (radiation, pollution, stress, and smoking), age, sex, fetal microchimerism, multiparity, and medication (alemtuzumab, interferon-alpha, ipilimumab, nivolumab, and pembrolizumab), or a combination these factors, have been implicated [11]. Alteration of the human intestinal microbiota, secondary to various infectious causes (viruses, bacteria, and parasites) and non-infectious ones (intestinal resections, drug treatments, eating habits, etc.), plays a relevant role in modulating thyroid function and predisposes individuals to autoimmune diseases [12,13].

AIT occurs secondary to the synthesis of antibodies against thyroid peroxidase, thyroglobulin, or TSH receptors (thyroid peroxidase, thyroglobulin, and thyrotropin receptor). Highlighting specific auto antibodies (anti-thyroid peroxidase (TPO), anti-thyroglobulin (TG), and anti-thyroid stimulating hormone receptor (TSH)) are diagnostic criteria for AIT [13]. Thyroid dysfunction, even subclinical, can influence the mechanisms of hormonal regulation in the body, including the pilosebaceous unit. The connection between the two has not been fully elucidated so far. On the other hand, the treatment of acne often involves oral medication with an intestinal dysbiotic role, a possible trigger for autoimmune diseases [13,14,15].

This study investigates the incidence of AIT in a group of acne patients, and determines the impact and influences of this pathology over the severity of acne through the disorders it produces in the body (namely hyper-/hypo-function of the thyroid), as well as the presence of specific antibodies. The results obtained may contribute to more frequent and correct diagnoses of subclinical AIT in this category of patients. As far as we know, there are much available published data that address this topic, specifically the aspects regarding the influence of AIT over the acne severity degree.

## 2. Materials and Methods

### 2.1. Study Design

This study included 236 patients diagnosed with acne in the dermatological office of Pelican Hospital and in few private medical offices of dermatology from Oradea, Romania, during January 2018–December 2020. The facts and figures were gathered, recorded, and analyzed. We considered the clinical and paraclinical data (form of acne, thyroid function, and antithyroid antibodies) and the demographic aspects like origin, age, and gender. Before beginning the research, all of the patients were questioned about their family and medical history concerning thyroid illnesses, and those that were following a treatment for a thyroid disorder were not included. None of the patients were following a treatment with systemic antibiotics, oral contraceptives, isotretinoin, or antiandrogens.

For the patients included in the study, the following thyroid hormone profiles were determined in order to assess AIT: thyroid-stimulating hormone (TSH), antithyroglobulin antibodies (anti-TG), and anti-thyroid peroxidase (TPO) antibodies. Patients were divided into two groups—170 patients with acne and AIT, and 66 patients with acne without thyroid dysfunction. Both groups were compared in terms of acne severity; moreover, in the AIT group, correlations were established between the severity of acne and thyroid dysfunction, as well as the values of antithyroid antibodies.

### 2.2. Clinical and Paraclinical Investigations

The dermatological diagnosis of acne was established on clinical criteria depending on the severity of acne, as follows:

Mild acne—characterized by the presence of some inflammatory lesions (papulopustular), non-inflammatory lesions, or both;

Moderate acne—described as mild scarring and incidental nodules, inflammatory lesions, or both;

Severe acne—characterized by nodules, extensive inflammatory lesions, or both, as well as injuries, moderate acne persisting even after 6-month therapy, and acne determining a serious psychological impact [16,17].

The diagnosis of AIT was established through paraclinical criteria, through the determination of the anti-thyroglobulin and anti-thyroid-peroxidase antibodies. The blood panels and the values for the thyroid antibodies and hormones (TSH, anti-TG, and anti-TPO) for all of the subjects were determined in the laboratory of Pelican Hospital. The samples were taken on an empty stomach and were processed on the same day. The reference range, established by the laboratory was 0.39–4.00 for women aged between 13–20 years and 0.40–4.00 for both women/men over 20 years; for anti TPO <35, and for anti-TG <115, regardless of age or sex. The antibody assays were negative or positive, and the results of the thyroid activity were classified as normal, hypofunction, or hyperfunction.

### 2.3. Statistical Analysis

IBM SPSS (Statistical Package for the Social Sciences) Statistics for Windows, Version 26, released 2019 (IBM Corp., Armonk, NY, USA) was used for the statistical analysis and graphical representation. Student’s and Chi-square tests were used to calculate the value of *p*. Statistical significance was considered for a value of *p* lower than 0.05.

The ANOVA unifactorial variant analysis was used to compare the observed means for n samples (n greater than 2), using the means (M_1_, M_2_, …, M_n_), standard deviations (DS_1_, DS_2_, …, DS_n_), and sample sizes (N_1_, N_2_, …, N_n_). The comparison of the means was performed using the parameter F of the Fisher test, for (n − −1) and (N − n) degrees of freedom. The difference between the means was considered statistically significant if the *p* value of the Fisher test was lower than 0.005.

In order to establish the type of post-hoc ANOVA analysis, for the cases with statistically significant ANOVA test results, the univariate dispersive analysis (Levene test) was used to test the dispersion homogeneity of the resulting samples. If the result of the Levene test was not statistically significant, the dispersions were considered equal and if otherwise, they were considered unequal. The Hochberg GT_2_ test, applicable to groups with different numbers of subjects and equal dispersions, was used as a post-hoc analysis test to compare the groups two by two.

A risk analysis was performed to identify the chance that the presence of certain hormones may be responsible for worsening acne in patients with TAI. If the link between these two factors could be proven, it could be considered that patients with a hormonal constellation would be more likely to develop severe forms of acne over time. According to the results of the logistic regression analysis, the association between the values of the odds ratios over unit, with a confidence interval higher than one and *p* values greater than 0.05, was interpreted as a major statistically significant risk. The Fischer exact formula was performed for calculating the *p* values cases of a chi square test.

## 3. Results

The incidence was determined by reporting the percentage of patients diagnosed with AIT to the total number of acne patients investigated during the study period. Of the 236 patients, 170 (72%) had elevated serum levels of antithyroid antibodies. The patients in the AIT group were statistically significantly older compared with group A (*p* < 0.001, t test). Severe acne was found to be significantly more common in the AIT group (*p* < 0.001 Chi-square). The demographic and clinical characteristics are presented in Table 1 and Table 2, which summarize the antibody values.

**Figure 1:**
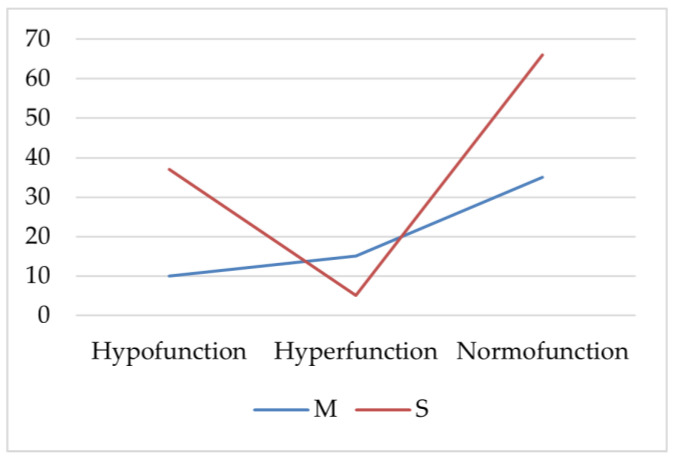
The frequency of severe and moderate acne in the autoimmune thyroiditis (AIT) group. M—moderate acne; S—severe acne.

AIT patients with hypofunction or normal thyroid function had severe acne (S) more frequently (*p* < 0.001, *p* = 0.002, Chi-square). Patients with thyroid hyperfunction more frequently had statistically significantly moderate acne (M; *p* = 0.025, Chi-square; Figure 1).

**Figure 2:**
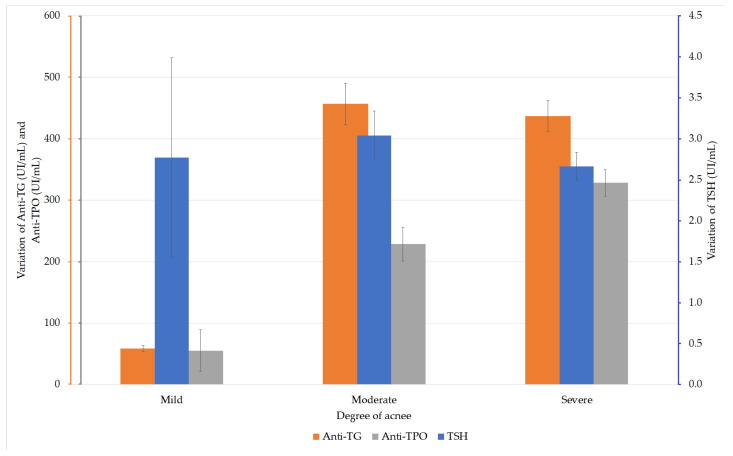
Variation of thyroid-stimulating hormone (TSH), anti-thyroglobulin (TG), and anti-thyroid peroxidase (TPO) values in patients with AIT and acne.

The TSH values were analyzed in the patients with acne. The ANOVA test did not reveal any statistically significant differences between the values in the three groups (F (2, 167) = 0.707, *p* = 0.494). Depending on the severity of acne, the analysis of anti-TG values in the acne patients did not reveal any statistically significant difference between these values (F (2, 167) = 2.231, *p* = 0.111 (ANOVA)). The analysis of the anti-TPO values according to the degree of acne revealed significantly higher values (F (2, 167) = 5.126, *p* = 0.007) in patients with severe acne compared with those with moderate acne (Figure 2).

The groups were considered to have equal dispersions, according to the analysis performed with the Levene test (*p* = 0.099). The post-hoc analysis performed by the Hochberg GT_2_ test showed a statistically significant difference only between the values of the groups with moderate and severe acne (*p* = 0.016; Table 3).

**Figure 3:**
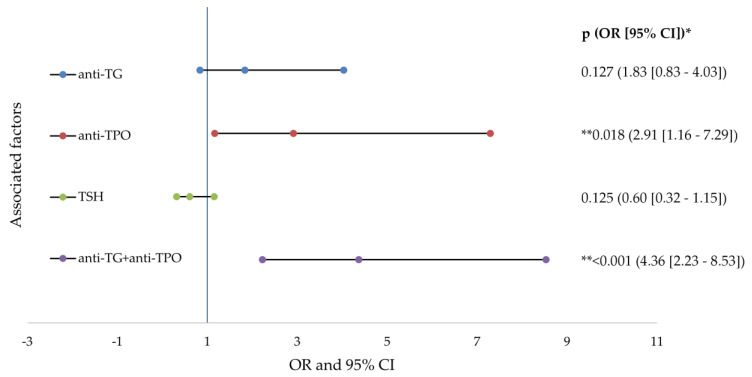
Forest plots of factors associated factors with severe acne (logistic regresion analysis). anti-TG—antithyroglobulin antibody; anti-TPO—thyroid peroxidase antibodies; TSH—thyroid stimulating hormone; OR—odd ratio; CI—confidence interval; *p* value—statistical significance; * logistic regression analysis; ** *p* values < 0.05.

The risk analysis indicated that the presence of increased anti-TPO (2.91 times) and the association with anti-TG (4.36 times) doubles the risk of developing severe acne in patients with AIT. All of the data studied in the risk analysis are presented in detail in Figure 3.

## 4. Discussion

The latest epidemiological investigations have demonstrated that there seems to be a rise in the number of patients with postadolescent acne, the disorder persists, and treatment is needed up until the mid-40 s, even though acne is usually considered a disease of adolescence [7]. So far, the factors that lead to postadolescent acne are not totally understood. Various environmental conditions (job-related, stress, photoexposure, and environmental conditions) are likely to contribute to hyperkeratinization and hyperseborrhea, which that are apparently common in postadolescent acne, despite the fact that it primarily targets androgens. Acne, a chronic inflammatory disease of the pilosebaceous unit, is influenced by multiple endocrine factors. In several chronic inflammatory skin disorders comprising chronic idiopathic urticaria and acne, thyroid autoimmunity was identified [18].

In recent years, in dermatological medical practice, increased incidence of AIT associated with moderate-severe acne has been observed in people over 20 years old, especially in females in the North-Western part of Romania. Because of the association of these two pathologies, this acne has proven to have a slower and longer lasting therapeutic response, compared with acne that has no associated hormonal dysfunction. Patients with severe acne associated with AIT require long-term systemic and local dermatological treatment compared with patients with severe acne without the associated hormonal problems. There were patients who discontinued dermatological maintenance treatment when they thought they had healed, and who had severe acne relapses. In Romania, patients with chronic diseases are not consistent with their treatment—they discontinue the treatments recommended by doctors when they consider and find that they are better, and for this reason many patients have frequent relapses—in this case, in the sense of worsening acne. Subjects with associated hormonal problems frequently have chronic acne with permanent dermatological maintenance treatment, and often need continuous hormone replacement therapy in order to keep their hormonal disorders within normal limits. Paraclinical investigations are mandatory in patients with moderate/severe acne over the age of 20 years old. In our study, there were patients who first presented with with acne vulgaris at the age of 40, with no history of acne up until that age, and in most cases, hormonal disorders were associated, most commonly AIT. This was the main reason our study investigated the incidence of AIT in people with chronic acne, and the correlation between the severity of the acne with thyroid imbalances caused by this pathology.

The pioneers in identifying that postadolescent women that suffer from acne presented elevated levels of thyroid autoimmunity vs. healthy subjects were Vergou and colleagues in 2012. The results of their research showed a statistically significant higher prevalence of anti-TG antibodies (25.2%) in the acne group compared with the control group. No significant differences were found in the prevalence of anti-TPO antibodies [7]. In 2017, Stewart and Bazergy determined a 24.5% prevalence of anti-TG antibodies, and 18% of the anti-TPO antibodies were found in adult women with acne, thus noting significantly higher values compared with the control group [19].

The results of the present study indicate increased incidence of AIT in the studied population with acne; 59.3% patients had high serum levels of anti-TG antibodies and 62.2% of anti-TPO antibodies. The observed differences may be caused by genetic factors, environmental conditions (i.e., iodine intake), or by the methods used for antibody dosing [13,14,20,21].

It can be stated that the etiology of AIT is multifactorial and is derived from the correlation of the actions between environmental/genetic factors; at the same time, the tendencies of the evolution of the prevalence, specifically of the incidence of AIT, were found to not be clear [22]. The usual practical experience confirms that the incidence of AIT is increasing, as also confirmed by some of the existing published data. However, it should be noted that tracking and comparing the actual patterns of the disorder is difficult and out of reach [23,24]

In addition, it is estimated that the thyroid antibody prevalence in the general population is somewhere between 10–12%, as seen in the published data [25]; moreover, different TPOAb positive rates were published in the National Health and Nutrition Examination Survey III (NHANES) [26], which considered that >10% of the studied adults were positive, namely for TPOAb (13% prevalence) and TgAb (11.5% prevalence). The study of Pedersen et al. also indicated (in Danish population) a 13.1% prevalence rate for TPOAb [27]. Furthermore, limited longitudinal studies exist that have researched the incidence of thyroid antibodies, such as the long-term (20-year) Whickham trial, which confirmed that 7% of men and 17% of women developed anti-thyroid antibodies [4,28,29,30,31,32]. Another study conducted and published by Li and colleagues reported that the cumulative incidence of TPOAb positivity was 2.81% over 5 years [33]. In addition, the conclusions of a study from 2019 in an urban area, considered an incidence of 23.22% AIT in healthy females (aged 20–30 years old being the most affected) with no previously diagnosed thyroid disease [34].

AIT presence was associated with a significantly higher age and significantly higher frequency of severe acne than in group A. In addition, in this study, women had statistically significant more frequent AIT than men (87.65% vs. 12.35%). The results are consistent with data from the medical literature, which state that females are more prone to autoimmune diseases, secondary to specific genetic or endocrine factors [35,36,37].

Within the AIT group, 47 patients presented thyroid hypofunction and 20 hyperfunction. Severe acne had a significantly higher frequency in patients with hypofunction and normal thyroid function compared with those with hyperfunction, which was dominated by moderate acne. The mean TSH values did not indicate significant differences in the severity of the pathology.

Anti-TG values did not significantly influence the severity of acne, while anti-TPO values were significantly higher in patients with severe acne.

In the present research the incidence of AIT was demonstrated by the risk analysis carried out, involving significant variations in acne development. The data analysis showed that the presence of increased anti-TPO (2.91 times) and the association with anti-TG (4.36 times) doubled the risk of developing severe acne in patients with AIT.

Some authors consider the psychosocial implications of acne as criteria for assessing the severity of acne, because they can, in some cases, be extremely severe (depression, anxiety, and suicidal tendencies). Because acne lesions appear on the visible skin and the onset is in adolescence, a period of increased vulnerable psychological status, acne can bring changes in psychosocial behaviour (low self-esteem, social isolation, difficulties in family relationships, feelings of frustration, and affecting professional activities) [38].

The treatment of acne is a very complex one and is established individually for each patient, depending on the severity of the acne and the associated conditions of patient. Treatment aims mainly to combat the main pathogenetic mechanisms that determine and maintain the disease, but it is important to initiate it early, in order to limit the psychosocial damage for the patients [39]. According to our study, acne may be considered to have a collaborative layout, indicating an endocrinological contribution, and we thus recommended routine assessment of thyroid antibodies for women with postadolescent acne. A low reference limit needs to be implemented for mild impairments and, critically, assessing the antibodies remains important, from the start of normal TSH screening. This interconnection should be emphasized in extensive groups, and the genetics potentially linking these two disorders should be demonstrated by subsequent research.

This study’s limitations are as follows: the first would be that we measured only TSH, with FT4 not being considered in order to investigate the thyroid function. Second, although detailed information of the patients’ history was obtained at the time of examination, the use of other medications (including thyroid related) and the presence of thyroid diseases (goiter) were self-reported. Moreover, it must be underlined that urinary iodine excretion was not measured, even though this research was conducted in an iodine sufficient country [40]. The following are among the strengths of the study: unlike other studies that investigate the severity of acne in patients with antithyroid antibodies compared with a control group (without acne), this study investigates the incidence of AIT in patients with acne and evaluates its severity, compared with both patients with acne without AIT, and depending on the presence and levels of TG and TPO (separately and together, in the group with AIT). As far as we know, our research is the only study on this topic that has included patients of both sexes. The results we obtained provide valuable information for clinicians in order to improve their guidance and management of the treatment of severe chronic acne.

## 5. Conclusions

The results of this study indicate an association between the changes in the thyroid function, presence of antithyroid antibodies, and severity of acne in young patients. Assessing the thyroid antibodies in adult patients with acne should be considered by clinicians, because positive results lead to treating a higher number of subjects, as it is indicated by the current study outcomes.

## Figures and Tables

**Table 1:** Demographic and clinical characteristics.

Characteristics	AIT + Acne (*n* = 170)		Acne (*n* = 66)		*p* Value
	No.	%	No.	%	
Gender					
Women	149	87.65	38	57.58	0.012 *
Men	21	12.35	28	42.42	<0.001 *
Age (years)					
12–21	26	32.05	52	67.54	0.002 *
>21	144	87.34	14	9.22	<0.001 *
Average	29 ± 8 years		18 ± 6 years		<0.001 **
Diagnostic					
Mild acne	2	11.11	16	88.89	<0.001 *
Moderate acne	60	63.16	35	36.84	0.010 *
Severe acne	108	87.80	15	12.20	<0.001 *
Thyroid function					
Hyperfunction	20	11.76	0	0	<0.001 *
Normal function	103	60.59	66	100	
Hypofunction	47	27.65	0	0	
Antibodies					
Anti-TG					<0.001 *
<115 UI/mL	30	17.65	66	100	
≥115 UI/mL	140	82.35	0	0	
Anti-TPO					<0.001 *
<35 UI/mL	23	13.53	66	100	
≥35 UI/mL	147	86.47	0	0	
TSH					<0.001 *
<0.39 (0.4) UI/mL	20	11.76	0	0	
0.39 (0.4)–4 UI/mL	103	60.59	66	100	
≥4 UI/mL	47	27.65	0	0	

* Chi-square test; ** t test.

**Table 2:** Antibodies values.

Characteristics	AIT + Acne (*n* = 170)		Acne (*n* = 66)		*p* Value
	M (UI/mL)	SD (UI/mL)	M (UI/mL)	SD (UI/mL)	
Anti-TG					
Total	439.4	264.9	19.29	16	<0.001 *
Mild acne	58.50	6.36	12.62	11.92	<0.001 *
Moderate acne	456.57	259.20	20	14.82	<0.001 *
Severe acne	436.85	266.22	23.8	20.67	<0.001 *
Anti-TPO					
Total	289.8	224.6	8	4.59	<0.001 *
Mild acne	55.30	47.65	5	3.99	<0.001 *
Moderate acne	228.63	210.70	8	4.46	<0.001 *
Severe acne	328.12	224.60	10	4.15	<0.001 *
TSH					
Total	2.79	1.97	2.11	0.79	<0.001 *
Mild acne	2.77	1.72	1.81	0.84	0.183 *
Moderate acne	3.03	2.29	2.36	0.67	0.095 *
Severe acne	2.79	1.97	1.85	0.85	0.071 *

* t test.

**Table 3:** Multiple comparisons of the anti-TPO values in patients with AIT and acne (Hochberg GT_2_ test).

Type of Acne	Degree of Acne	Average Difference (I–J)	Average Standard Error	*p*	95% Confidence Interval	
					Lower Limit	Upper Limit
Mild	Moderate	−173.327	157.645	0.614	−553.42	206.77
	Severe	−272.820	156.511	0.228	−650.18	104.54
Moderate	Mild	173.327	157.645	0.614	−206.77	553.42
	Severe	−99.494 *	35.314	0.016	−184.64	−14.35
Severe	Mild	272.820	156.511	0.228	−104.54	650.18
	moderate	99.494 *	35.314	0.016	14.35	184.64

*p*—statistical difference; * mean difference is significant for *p* < 0.05.

## Data Availability

Data supporting reported results can be found in the archive of the Pelican Hospital of Oradea, Romania.
